# The Role of Adults in Poliovirus Transmission to Infants and Children

**DOI:** 10.9745/GHSP-D-23-00363

**Published:** 2024-04-29

**Authors:** T. Jacob John, Dhanya Dharmapalan, Robert Steinglass, Norbert Hirschhorn

**Affiliations:** a Christian Medical College, Vellore, India.; b Apollo Hospitals, CBD Belapur, Navi Mumbai, India.; c Independent immunization advisor, Marshall, NC, USA.; d Independent consultant, Minneapolis, MN, USA.

## Abstract

We draw attention to a neglected aspect of poliovirus transmission—the likely role of adults in sustaining transmission—which has important policy and practical implications for addressing the perplexing phenomenon of continued virus circulation.

## Key Messages

- Adults who are immune and protected from polio nevertheless can, and do, get reinfected with wild polioviruses and become part of transmission chains.
- Circulating vaccine-derived polioviruses are wild-like, and adults may get infected and continue transmitting them to children.
- As the primary source for reinfection in adults are first-time infected infants and children, additional doses of inactivated poliovirus vaccine for children will help to interrupt all transmission chains and accelerate polio eradication.

## INTRODUCTION

Every communicable disease results from 3 events: pathogen amplification, pathogen transmission, and host-pathogen interaction.^1^ While disease is overt, pathogen amplification and transmission are covert, discernible through epidemiological investigation. Disease can be prevented in the individual by immunization, resulting in drastic alteration of host-pathogen interaction. For disease elimination/eradication, a vaccination program’s strategy must be designed not only to protect all individuals from disease but also to interrupt all transmission chains.^2^

In 1988, poliomyelitis (polio) was targeted for eradication by the year 2000.^3^ Wild poliovirus (WPV) is an exclusive human pathogen, amplification occurring in infected individuals. However, the transmission dynamics of WPV have not been adequately investigated to explain the main chains of transmission.

Many global polio experts believe in the superiority of live oral poliovirus vaccine (OPV) over inactivated polio vaccine (IPV) for stopping the spread of poliovirus in the community,^4^^,^^5^ reasoning that only OPV induces secretory immunoglobulin A (IgA)-mediated mucosal immunity. Nonetheless, the intriguing phenomenon of continued virus circulation—WPVs until 2012 and overwhelmingly circulating vaccine-derived polioviruses (cVDPVs) since then— despite years of heavy vaccination pressure with OPV demands an explanation.

All immunization activities of the Global Polio Eradication Initiative are in children aged younger than 5 years. In low- and middle-income countries (LMICs), they get many doses of OPV, and only in some countries, just 1 or 2 doses of IPV. Do only children with first infection transmit WPV? Or do immune individuals—immune due to past WPV infection or immunization with OPV—get reinfected and participate in transmission? If immune individuals get reinfected and transmit WPV, mucosal immunity’s ability to inhibit transmission cannot be robust or long-lasting.

## DO REINFECTED IMMUNE ADULTS HAVE A ROLE IN POLIOVIRUS TRANSMISSION?

A set of natural experiments—long-distance importations of WPVs from polio-endemic to non-endemic countries across continents—were available for investigation for over 2 decades to shed light on who the vectors of transmission are: only first-time infected infants/children or reinfected immune adults also? During 2003–2006, there were 68 separate importation episodes into 24 previously polio-free countries.^6^ In 2021, WPV-1 was imported from Pakistan into Malawi, most probably carried by adults without signs of infection and traveling for trade/business.^7^ In 2022, a lineage of cVDPV type 2 was detected in sewage surveillance in London, Jerusalem, and New York in quick succession.^8^ It was highly probable that adult travelers acted as carriers.^8^

Bulgaria had an importation of WPV-1 in 2001, and the episode was investigated by the National Centre for Infectious and Parasitic Diseases, Sofia.^9^ The outbreak started in the port city of Bourgas in March, lasted until May, and was interrupted by 2 national mass immunization campaigns using trivalent OPV. The origin of the virus, determined by molecular tests, was India. Four cargo ships with 34 crew members (34/67=51%) of Indian nationality had docked in the harbor during February and March 2001.^9^ It is reasonable to assume that Indian sailors were the vector of transmission between India and Bulgaria.^9^ There was no information about the time lapse between their departure from India and arrival in Bourgas. Perhaps an infected sailor transmitted infection to 1 or more crew members, enabling virus persistence during travel. It is very likely that most, if not all, long-distance WPV and cVDPV importations were through reinfected adults who were themselves immune and not at risk of paralysis.^10^

In July 2007, an Australian traveler aged 22 years returned from Pakistan to Australia and soon developed polio.^11^ In 2022, an adult in New York without a history of foreign travel developed polio due to cVDPV.^12^ Because the case-to-infection ratio is only a fraction of 1 (as noted later), for every adult with the disease, many more would have been infected because poliovirus does not selectively enter only immunity-naïve adults.

There is consensus that poliovirus is highly contagious and transmitted person-to-person during ordinary social interactions.^13^ In non-immune individuals, polio develops in less than 1% of first-time infected persons, mostly children, for a case-to-infection ratio of <0.01.^13^ More than 99% of all first infections are without paralysis and, hence, go unrecognized.

In LMICs, where the polio eradication efforts are ongoing, polio disease and polio immunization, both routine and supplementary, continue to be confined to children aged younger than 5 years. In pre-eradication times, polio began to occur in infants aged 4 to 5 months as soon as maternal antibody protection waned, reached the median age of 12–13 months, and saturated children by age 5 years.^14^^,^^15^ Due to relatively high birth rates, children aged younger than 5 years constituted 10%–15% of the total population. A graph of this age profile would illustrate that exposure to infection is a function of age, with exposure at a fairly constant rate, largely unaffected by extrinsic environmental factors. At any given time, about two-thirds of children aged younger than 5 years were under the curve—already infected and immune. First infections in the future were confined to a third of children aged younger than 5 years, accounting for only about 3%–5% of the total population, dispersed among 95% to 97% immune individuals. Immunity naïve children could not have sustained transmission exclusively among themselves. Transmission chains of WPV had to include individuals with reinfection and contagiousness, despite immunity—did they include adults also?

Transmission chains of poliovirus had to include individuals with reinfection and contagiousness, despite immunity—did they include adults also?

There is a notable difference between high-income countries and LMICs regarding sociocultural behavior about child-rearing practices. Unlike in high-income countries, in LMICs, infants and toddlers are not distanced from adults, partly due to space constraints in houses. Infants are often carried by mothers to workplaces, shops, and markets. Opportunities abound for physical proximity among adults and young children. The probability/frequency of reinfected adults acting as links in WPV transmission chains for infection reaching infants and young children remains to be investigated.

Children immunized with OPV and protected from polio can act as links in the WPV transmission chains.^16^^,^^17^ Reinfection with WPV has actually been demonstrated in India.^18^ A poliovirus transmission modeling analysis was published in 2012 to understand the difficulties faced by the Global Polio Eradication Initiative, which has been struggling to achieve polio eradication far beyond the original target year of 2000, relying on OPV.^19^ The authors concluded that, although systemic immunity against paralysis is lifelong, mucosal immunity against reinfection wanes over time.^19^ Reinfections, including in adults, are also contagious—the prevailing notion that only children aged younger than 5 years or only immunity-naïve individuals transmitted polioviruses is erroneous.^19^

## A PROPOSED INTERVENTION TO INTERRUPT POLIOVIRUS TRANSMISSION

The IPV is known to boost mucosal immunity induced by WPV or vaccine virus infection far better than OPV.^13^^,^^19^^,^^20^ Mayer et al. considered the advantage of giving a dose of IPV to all adults in countries with endemic polio due to WPV or cVDPVs to expedite the completion of polio eradication but realized that it would not be a practical proposition.^19^ They argued against offering OPV to all adults for fear of the emergence of new lineages of VDPVs.^19^

We know that first infection results in maximal virus shedding. Transmission chains start with infants and children aged younger than 5 years who serve as a source of infection in adults. Adults may continue with many silent chains of transmission. Disease can occur only in those who are not immune—infants and young children in endemic countries or non-immune children and adults in far-away countries after introduction. With this new insight, we have an urgent need and opportunity to try to interrupt all transmission chains.

If additional doses of IPV-containing vaccine are given to children aged younger than 5 years at well-chosen age points, it is likely that the number of unseen transmission chains can be curtailed if not eliminated. Perhaps 2 doses, first at 12 months and the second at 24 months, may suffice for the purpose, but mathematical modeling, field investigations, and operational considerations can help to fine-tune the timing of the minimum number of IPV doses for optimum benefit.

In summary, we present strong circumstantial evidence for adults’ role in poliovirus transmission and propose that intervention to prevent it will expedite interrupting transmission of WPVs and cVDPVs in their respective endemic countries. We recommend that every future episode of importation be thoroughly studied to understand how virulent polioviruses are carried long distances.

## Notes

**Peer Reviewed**

**First published online:** April 1, 2024.
